# The Impact of Virtual Reality Simulation Training on Earthquake Preparedness Knowledge and Practices Among Rural Volunteers in Indonesia: Quasi-Experimental Repeated-Measures Study

**DOI:** 10.2196/74108

**Published:** 2025-10-31

**Authors:** Nyayu Nina Putri Calisanie, Tukimin bin Sansuwito, Regidor III Dioso, Linlin Lindayani

**Affiliations:** 1 School of Allied and Nursing Science, Lincoln University College, Petaling Jaya, Selangor, Malaysia; 2 Sekolah Tinggi Ilmu Keperawatan PPNI Jawa Barat, Jl Ahmad 4 No 32, Pamoyanan, Kec. Cicendo, Kota Bandung, Jawa Barat, 40173, Indonesia, 62 817 0780 777; 3 Department of Health Science, Lincoln University College, Petaling Jaya, Selangor D E, Malaysia

**Keywords:** virtual reality, disaster preparedness, earthquake training, rural volunteers, simulation-based training

## Abstract

**Background:**

Natural disasters, including earthquakes, threaten global sustainable development, causing significant loss of life, displacement, and economic damage. Indonesia, located in the Pacific Ring of Fire, faces frequent seismic events, highlighting the need for effective disaster preparedness. Traditional training methods often fall short in practical application, prompting the exploration of innovative tools like virtual reality (VR) simulations. VR offers immersive, scenario-based training, improving knowledge retention and response skills.

**Objective:**

This study evaluated the effectiveness of VR simulation training in improving earthquake preparedness knowledge and practical response skills among rural volunteers in Indonesia.

**Methods:**

This quasi-experimental research involved 400 rural volunteers who were evenly divided into 2 groups: an intervention group (n=200) trained using VR simulations and a control group (n=200) that received standard training. The VR training modules covered earthquake awareness, search and rescue operations, first aid procedures, and evacuation practices. Participants’ knowledge and practical skills were evaluated using the Earthquake Preparedness Knowledge Questionnaire and Earthquake Response Practical Skills Assessment at baseline, immediately after training, and at a 3-month follow-up. Data analysis used repeated-measures ANOVA and multiple regression.

**Results:**

Volunteers trained with VR demonstrated substantial improvements in both knowledge (*F*_2396_=45.32; *P*<.001) and practical skills (*F*_2396_=38.76; *P*<.001) compared with the conventional training group. Post hoc tests confirmed that these improvements remained consistent even after 3 months. Regression analysis indicated education level (*β*=0.32; *P*<.001) and age (*β*=−0.18; *P*=.02) significantly influenced VR training outcomes. After controlling for demographic factors, the VR intervention still significantly enhanced earthquake preparedness knowledge (*β*=6.23; *P*<.001) and practical response abilities (*β*=5.45; *P*<.001).

**Conclusions:**

VR simulation training significantly boosts earthquake preparedness knowledge and practical response skills among rural Indonesian volunteers, with enduring benefits. These findings support VR’s potential as a scalable, effective disaster preparedness tool in resource-constrained environments.

## Introduction

### Background

Natural and human-made disasters pose significant threats to global sustainable development, as outlined in the United Nations Sustainable Development Goals [1]. Earthquakes are one of the most catastrophic natural disasters, accounting for approximately 60% of disaster-related deaths and economic losses worldwide, impacting millions of individuals annually [2]. Earthquakes killed more than 10,000 people and displaced more than 1.5 million in 2022 alone [3]. Indonesia, part of the seismically active Pacific Ring of Fire, is particularly susceptible, with an average of 8260 seismic events recorded each year, of which approximately 200 cause major damages [4]. These disasters not only cause physical destruction—they can also have devastating implications for public health, economic stability, and social cohesion, particularly in resource-limited rural settings [5]. The 2018 Sulawesi earthquake and tsunami, which resulted in the loss of over 4300 lives and displaced more than 170,000 people [4], highlight the essential importance of effective disaster preparedness strategies.

Natural disaster preparedness is an important initiative to prevent loss and damages. It involves understanding health and safety measures, crafting emergency responses, and making sure these measures are put into practice [6]. Communities with strong preparedness measures see fewer deaths and recover more quickly [7]. For example, after Japan’s earthquake in 2011, areas with robust disaster plans showed noticeably lower death rates [8]. Likewise, households in the United States that had emergency kits were better prepared when Hurricane Harvey struck and fared better with respect to injuries and trauma [9]. In Nepal, communities where disaster education and early warning systems were accessible showed increased resilience in the face of the 2015 Gorkha earthquake, facing lower mortality rates[10]. In Turkey, research also showed that municipalities with well-developed plans were in a better position to mitigate the impact of the Elazığ earthquake in 2020 [11]. In contrast, Indonesia’s rural areas, which often lack adequate disaster training, were disproportionately negatively affected by similar events [12]. Risk reduction programs implemented at the local level have been shown to lessen both fatalities and property damage during earthquakes [13]. A specific goal, such as these steps toward disaster preparedness, would help mitigate risk, enhance response, and build community resilience [14].

Disaster preparedness efforts rely heavily on volunteers, particularly in rural Indonesia, where they double as first responders and educators [15]. However, their effectiveness is contingent on adequate training, which is often lacking [16]. A 2020 study from the Philippines showed that trained volunteers dramatically improved response times during Typhoon Vamco, but there was an absence of structured training programs [17]. Despite this, a 2021 study in India found that even motivated volunteers were at a disadvantage when responding to floods due to limited training resources[18]. In Egypt, volunteers were effective in preparing for an earthquake but did not have the necessary technical skills [19,20]. A study from Turkey conducted in 2022 showed that regularly trained volunteers are more effective at providing emergency responses [21]. In Indonesia, a 2021 study confirmed the significance of volunteers in mobilizing communities but highlighted shortcomings in their first aid and rescue skill sets due to a lack of training that addressed rural specificities [22]. Although motivated, volunteers frequently lack adequate technical skills, practical experience, and context-sensitive education—factors that compromise disaster response effectiveness [21,22].

Traditional training sessions for improving volunteer preparedness, including workshops and drills, often do not engage participants or accurately replicate real-life disaster situations [23]. Although these strategies enhance theoretical understanding, many participants often fall short in translating this understanding into practice when faced with real-life emergency conditions. This has also been reported with disaster preparedness workshops, which increase awareness but fail to prepare people for the stress and complexity of actual disasters [24]. Earthquake drills and education efforts similarly increase procedural awareness but lack the urgency and unpredictability of real events, thereby limiting their effectiveness for practical skills and confidence [25,26]. These didactic methods often lack interactivity and contextual immersion, which are crucial for skill mastery and behavioral readiness. Recent research has called for the integration of more dynamic and experiential learning platforms, such as scenario-based or immersive simulation training, to close this preparedness gap [27,28].

Virtual reality (VR) has been developed as a novel tool for training disaster preparedness through realistic immersive simulations [28,29]. It also improves knowledge retention and practical skills by allowing users to practice disaster scenarios in a controlled setting [30]. Research shows that these VR simulations significantly enhance disaster preparedness among various groups, from health care workers to students. For example, research has demonstrated that VR training improves participants’ ability to respond to earthquakes, navigate decision-making, and increase confidence in performing tasks, with VR participants exhibiting more rapid response times than traditional training participants [31]. Likewise, VR simulations for tsunami preparedness have improved participants’ situational awareness and their ability to navigate complex environments [32-34]. Despite this promise, the scalability, cultural relevance, and effectiveness of VR interventions remain underexplored in low- and middle-income countries, especially among community-based volunteer responders in high-risk, low-resource environments like Indonesia.

### Objective

This study aimed to evaluate the feasibility, cultural adaptability, and impact of a VR-based disaster preparedness training intervention tailored for community disaster response volunteers in rural Indonesia. By leveraging immersive digital technology in a resource-limited setting, this research contributes to the growing field of digital public health interventions. The study offers novel insights into how VR can be used to enhance disaster readiness, inform scalable training strategies, and improve community resilience in low- and middle-income countries.

## Methods

### Study Design

This research used a quasi-experimental repeated-measures design to assess the effectiveness of VR simulation training in enhancing earthquake preparedness knowledge and practical skills among rural volunteers in Indonesia. The study compared 2 groups: an intervention group that received VR-based training and a control group that participated in traditional disaster preparedness training. Data were collected at 3 stages: before the intervention (baseline), immediately after the training, and 3 months postintervention to evaluate knowledge retention and skill application.

### VR Development and Validation

The VR simulation focused on enhancing earthquake preparedness and response capabilities while providing an immersive and interactive industrial experience. This involved content validation, technical creation, and pilot-testing. Content was based on international disaster preparedness standards, including instructions from the World Health Organization, the Federal Emergency Management Agency, and Indonesia’s National Disaster Management Authority.

Developed in collaboration with disaster response experts, emergency medical professionals, and VR software engineers, the curriculum was designed to be as accurate and realistic as possible. A 2-round expert validation was conducted using the Delphi method with a panel of 10 disaster response experts. Experts then reviewed the content for accuracy, relevance, and feasibility, which together resulted in iterative refinements. The context validity index of the final VR module was 0.92, confirming its validity. Unity 3D was used to develop and program the simulation in C# to achieve high-quality graphics and interaction in the VR simulation.

**Figure 1.**
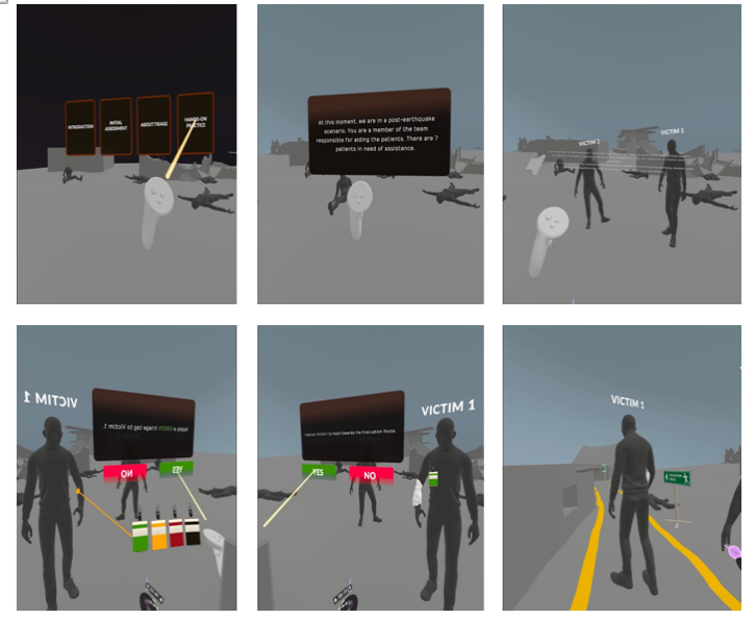
Prototype of virtual reality disaster simulation training.

The simulation was tailored for Meta Quest 2 VR headsets, which feature full head-tracking, gesture recognition, and real-world movement in the virtual space. Simulation highlights featured a 360° earthquake scenario simulating a high-magnitude event; participants determining appropriate responses in real time; interactive drills on triage, first aid, and evacuation; and haptic feedback and spatial audio to enhance sensory immersion and realism (Figure 1).

The VR module was beta-tested with 10 emergency responders, who gave feedback on usability and realism. Adjustments were made to improve navigation, task difficulty, and audiovisual effects. The training program consisted of five 45-minute sessions delivered over 2 weeks (Table 1).

**Table 1.** Virtual reality training module.

Session	Content
Session 1: Initial Assessment and Orientation	Pretest to assess baseline knowledge and skills Introduction to the virtual reality system and interaction mechanics
Session 2: Earthquake Awareness and Triage Simulation	Immersive earthquake scenario with collapsing structures and injured victims Training on the START (Simple Triage and Rapid Treatment) protocol for triage and victim prioritization Interactive decision-making exercises
Session 3: Search and Rescue Simulation	Navigation through a damaged environment to locate trapped individuals Training on safe movement in unstable structures and debris clearance Teamwork and communication exercises with virtual team members
Session 4: First Aid and Medical Assistance	Hands-on practice of cardiopulmonary resuscitation, wound care, and fracture immobilization Scenario-based responses to victims with varying injury severity Performance feedback after each task
Session 5: Safe Evacuation and Postearthquake Response	Practice of evacuation procedures and identification of safe routes Training on postearthquake hazards like aftershocks and fires Final assessment and feedback on overall progress

The control group received typical training, including lecture sessions, demonstrations, and group discussions without VR integration. To ensure the VR training program effectively improved learning outcomes, the following validation measures were implemented. For pretraining and posttraining assessments, participants completed the questionnaires at baseline, immediately postintervention, and at a 3-month follow-up. A posttraining evaluation survey was administered, measuring user satisfaction, perceived realism, and ease of use. The VR system recorded decision accuracy, response time, and task completion rates for each participant, allowing for quantitative analysis of skill improvements.

### Sample

Eligible participants were rural volunteers aged 18 years or older with no prior formal earthquake preparedness training. Additional inclusion criteria included normal or corrected vision and a commitment to attend all training sessions. Exclusion criteria included a history of motion sickness, medical conditions limiting physical activity, or incomplete participation.

The study enrolled 400 participants, evenly split between the intervention (n=200) and control (n=200) groups. The sample size was determined using G*Power analysis, with an effect size of 0.5 [35], power of 0.95, and α level of .05. Participants were recruited via convenience sampling from earthquake-prone rural areas in Indonesia to ensure demographic diversity.

### Instruments

Sociodemographic characteristics included questions on age, sex, education level, type of housing, work status, formal education, and perception of web-based earthquake preparedness education.

The questionnaire was designed to assess knowledge, perception, and practice regarding earthquake preparedness among Nepalese immigrants residing in Japan [36]. The questionnaire is divided into 3 sections. The section on earthquake preparedness general knowledge and practice (23 items) uses a 3-point Likert scale: yes (1 point), no (1 point), don’t know/not applicable (0 points). Example items include “Do you think your building is more vulnerable to earthquake damage?” “Do you know the evacuation center near your home?” and “Have you prepared an emergency bag?” The total score ranges from 0 to 23, with higher scores indicating better preparedness. The section on the effectiveness of the educational intervention (15 items) uses a true/false format: correct answer (1 point) and incorrect answer (0 points). Example items for this section include “In an earthquake, you should move to an open area” and “You should not use objects with fire hazards during the earthquake.” The total score ranges from 0 to 15, with higher scores indicating better understanding of the intervention. For this questionnaire, Cronbach α=.92, indicating high internal consistency [31].

The instrument first underwent an extensive adaptation process per the World Health Organization protocols on the translation and adaptation of instruments [37] for rural Indonesian participants to ensure cultural and contextual fit. The forward translation by two independent bilingual experts (native Indonesian speaker, English fluency) was reconciled by the bilingual experts for discrepancies. A third bilingual expert who had no previous role in the translation independently conducted a backward translation. Indonesian translation was then evaluated by a group of experts on disaster preparedness and community health for content relevance, conceptual equivalence, and construct validity in the rural Indonesian context. Lastly, the adapted version was piloted in 30 people from a demographically similar population in rural Indonesia. This included feedback from the pilot and whether terms were ambiguous or culturally appropriate. The Cronbach α (0.86) of the pilot study indicated good internal consistency and reliability in the setting of Indonesia. This extensive adaptation process lends support to the validity and applicability of the instrument in the context of rural Indonesian communities.

### Procedure

The recruitment started with outreach to rural community organizations and disaster volunteer networks in Lembang District, West Java, Indonesia. Volunteer recruitment posters were sent via several channels, including through health posts, community centers, and WhatsApp groups for volunteers. Participants were placed into either the intervention group or the control group once they had completed the enrollment process. The collection of baseline data took place immediately after the assignment of students to groups. The questions were constructed, and the participants answered them. While the control group got normal disaster preparation training in the form of lectures, demonstrations, and group discussions, the intervention group took part in the VR simulation training program. The VR training was carried out in a room that was specifically designated for that purpose, and it was supervised by research assistants who had received training in VR facilitation. Over the course of 2 weeks, there were 5 VR sessions, each of which lasted for 45 minutes. Over the course of the same time period, participants in the control group participated in 5 standard training sessions that were about the same length of time. Participants in both groups were required to retake the knowledge questionnaire and skills evaluation immediately after the conclusion of the final training session. In addition, participants in the intervention group were asked to fill out a posttraining evaluation survey to investigate their levels of satisfaction, perceived realism, ease of use, and potential discomforts associated with VR. Objective performance indicators, such as decision-making accuracy, reaction times, and job completion rates, were automatically acquired by the VR system and recorded automatically. After 3 months had passed since the intervention, each participant was called individually to complete a follow-up knowledge exam and to retake the checklist of practical abilities. The collection of data for both groups was carried out in person in community centers, which ensured that the assessment contexts were consistent. In order to ensure that participants were retained for as long as possible, follow-up assessments were planned in a flexible manner, and several reminders were sent out via WhatsApp messaging and phone calls.

### Data Analysis

Data were analyzed using SPSS (version 25; IBM Corp). Descriptive statistics were performed to summarize participant demographics and baseline measures. Repeated-measures ANOVA was used to compare the change in earthquake preparedness knowledge and practices from baseline to 3-month follow-up between the intervention and control groups. Assumptions for repeated-measures ANOVA (ie, normality and sphericity evaluated with the Mauchly test) and homogeneity of variances were checked before analysis. Greenhouse–Geisser corrections were applied in case of sphericity violation. A Bonferroni post hoc analysis was performed to determine between which time points any significant differences occurred. Then, we conducted a multiple regression analysis aimed at determining the relationship of the demographic variables (age, sex, education level) with the effectiveness of the VR intervention. Assumptions of linear regression (linearity, independence of errors (using the Durbin-Watson test), homoscedasticity, multicollinearity (using variance inflation factor), and normality of residuals were tested and fulfilled. A mixed-effects model was used to account for both fixed effects (intervention) and random effects (participant-level variability). The model included time and group as fixed effects and participant ID as a random intercept to account for within-subject correlations over time. Model fit was assessed using Akaike information criterion and Bayesian information criterion. Furthermore, to ensure the adequacy of the models, checks included residuals versus fitted, Q-Q plots, and standardized residuals. Effect sizes (partial η^2^ for ANOVA and standardized beta coefficients for regression) and 95% CIs were reported for a more intuitive grasp of the magnitude and precision of the effects. *P*<.05 two-tailed was accepted as statistically significant.

### Ethical Considerations

The study was approved by the institutional review board of STIKep PPNI Jawa Barat (III/098/KEPK/STIKep/PPNI/Jabar/III/2024) on March 4, 2024. All human participant procedures were in accordance with the ethical standards of the institutional research committee and with the 1964 Helsinki declaration and its later amendments. All subjects received information regarding the aims of the study, procedures, and risks and benefits in writing. All participants provided informed consent before being included in the study. Participants were informed that all information provided would be treated anonymously and securely. Identifiable information was accessible by the research team only. Participants participated voluntarily and had the opportunity to withdraw from the study at any time without consequence. No compensation was provided to participants.

## Results

### Participant Demographics

This study enrolled 400 participants, evenly distributed between the intervention group (n=200) and the control group (n=200), from March to May 2024. At the baseline assessment, both groups demonstrated similar demographic characteristics (Table 2). The predominant age range among participants was 18‐30 years, with 90 of 200 participants (45%) in the intervention group and 85 of 200 participants (42.5%) in the control group. Gender distribution was nearly equal, with 104 participants (52%) female in the intervention group and 101 participants (50.5%) female in the control group. A majority of participants had completed secondary education, reported by 141 participants (70.5%) in the intervention group and 136 participants (68%) in the control group. Most participants lived in single-family homes, with 130 participants (65%) in the intervention group and 135 participants (67.5%) in the control group. Employment rates were high across both groups, with 150 participants (75%) employed in the intervention group and 145 participants (72.5%) in the control group. Regarding prior disaster education, 120 participants (60%) in the intervention group and 116 participants (58%) in the control group had previously received formal education on disaster preparedness. Furthermore, a large proportion of participants viewed web-based earthquake preparedness education as beneficial, with 170 participants (85%) in the intervention group and 165 participants (82.5%) in the control group. No statistically significant differences were found between the two groups concerning age, gender, education level, housing type, employment status, prior disaster education, or perception of web-based earthquake preparedness training (*P*>.05).

**Table 2.** Demographic characteristics of participants (N=400).

Characteristic	Intervention group (n=200), n (%)	Control group (n=200), n (%)	*P* value
Age (years)			.45
18‐30	90 (45)	85 (42.5)	
31‐40	40 (20)	45 (22.5)	
41‐50	50 (25)	55 (27.5)	
>50	20 (10)	15 (7.5)	
Sex			.78
Male	96 (48)	99 (49.5)	
Female	104 (52)	101 (50.5)	
Education level			.62
Primary	30 (15)	35 (17.5)	
Secondary	141 (70.5)	136 (68)	
Tertiary	29 (14.5)	29 (14.5)	
Type of housing			.58
Single-family	130 (65)	135 (67.5)	
Multifamily	70 (35)	65 (32.5)	
Work status			.67
Employed	150 (75)	145 (72.5)	
Unemployed	50 (25)	55 (27.5)	
Formal education			.71
Yes	120 (60)	116 (58)	
No	80 (40)	84 (42)	
Perception of web-based education			.52
Useful	170 (85)	165 (82.5)	
Not useful	30 (15)	35 (17.5)	

### Primary Outcomes: Earthquake Preparedness Knowledge and Practices

Table 3 presents the results of repeated-measures ANOVA for earthquake preparedness knowledge and practices across 3 time points: baseline, postintervention, and 3-month follow-up. There was a significant interaction effect between group (intervention vs control) and time for both knowledge and practices. For earthquake preparedness knowledge, a significant main effect of time and group was found (*F*_2196_=45.32; *P*<.001; partial η²=0.316, 95% CI 0.231-0.392), indicating a large effect size. Post hoc Bonferroni comparisons showed that the intervention group experienced a significant increase in knowledge from baseline (mean 12.34, SD 2.45) to postintervention (mean 18.56, SD 1.89) and maintained improvements at follow-up (mean 17.89, SD 2.01), whereas the control group showed minimal changes.

For earthquake preparedness practices, the repeated-measures ANOVA also yielded a significant interaction (*F*_2196_=38.76; *P*<.001; partial η²=0.283, 95% CI 0.198-0.360), again representing a large effect. The intervention group’s practices improved significantly from baseline (mean 10.23, SD 1.98) to postintervention (mean 15.67, SD 1.45) and remained elevated at follow-up (mean 14.89, SD 1.50). In contrast, the control group exhibited only minor improvements.

**Table 3.** Repeated-measures ANOVA for earthquake preparedness knowledge and practices.

Variable and time point		Intervention group, mean (SD)	Control group, mean (SD)	*F* test	*P* value	Partial η²
Knowledge				45.32 (2196)	<.001	0.316
	Baseline	12.34 (2.45)	12.10 (2.30)			
	Postintervention	18.56 (1.89)	13.45 (2.10)			
	Follow-up	17.89 (2.01)	12.89 (2.15)			
Practices				38.76 (2196)	<.001	0.283
	Baseline	10.23 (1.98)	10.10 (1.85)			
	Postintervention	15.67 (1.45)	11.23 (1.56)			
	Follow-up	14.89 (1.50)	10.89 (1.60)			

### Secondary Analysis: Post Hoc Comparisons With Bonferroni Corrections

To pinpoint specific points of improvement, post hoc analyses using Bonferroni corrections were conducted. In the intervention group, significant progress in knowledge and skills was observed from baseline to immediately after the intervention (*P*<.001) and from baseline to follow-up (*P*<.001). However, no statistically significant differences were noted between postintervention and follow-up assessments (*P*>.05), suggesting that the benefits of VR training were maintained over time. Conversely, the control group showed no meaningful changes across any time points (*P*>.05; Table 4).

**Table 4.** Post hoc analyses with Bonferroni corrections.

Variable and comparison		Intervention group (*P* value)	Control group (*P* value)
Knowledge			
	Baseline versus postintervention	<.001	.12
	Baseline versus follow-up	<.001	.15
	Postintervention versus follow-up	.06	.89
Practices			
	Baseline versus postintervention	<.001	.10
	Baseline versus follow-up	<.001	.13
	Postintervention versus follow-up	.08	.92

### Predictors of Intervention Effectiveness

A multiple regression analysis was performed to explore the impact of demographic variables (age, gender, and education level) on the effectiveness of VR training. Results revealed that education level (*β*=0.32; *P*<.001) and age (*β*=−0.18; *P*=.02) were significant predictors of improvements in earthquake preparedness knowledge and practical response, while gender had no significant influence (*β*=0.05; *P*=.45; Table 5).

**Table 5.** Multiple regression analysis for predictors of virtual reality intervention effectiveness.

Predictor	β coefficient	Standard error	95% CI	*P* value
Age	−0.18	0.08	−0.34 to −0.02	.02
Gender (female)	0.05	0.07	−0.09 to 0.19	.45
Education level	0.32	0.06	0.20 to 0.44	<.001

### Adjusted Intervention Effects: Mixed-Effects Modeling

To control for potential confounding factors, a mixed-effects model was applied, incorporating random effects for participant variability and fixed effects for the intervention. This model demonstrated a strong fit, with an Akaike information criterion of 1256.34 and a Bayesian information criterion of 1289.45. Findings reaffirmed the substantial impact of VR training on earthquake preparedness knowledge (*β*=6.23, SE=0.45, 95% CI 5.35-7.11; *P*<.001) and practical response skills (*β*=5.45, SE=0.38, 95% CI 4.70-6.20; *P*<.001), even after adjusting for demographic variables.

## Discussion

### Principal Findings

This study demonstrates that VR simulation significantly enhances earthquake preparedness knowledge and practices among rural volunteers in Indonesia, with improvements sustained over a 3-month period. These findings underscore VR’s potential as a scalable, low-cost, and context-sensitive training tool, especially for rural settings where access to conventional disaster preparedness programs is limited.

Our findings reinforce and expand prior work establishing the advantages of immersive technologies in disaster training [38], which found that VR-based training offers realistic and immersive environments that help facilitate knowledge retention as well as skill acquisition—VR-based training is thus said to be superior to traditional tabletop exercises. Other studies [25,28,39] also acknowledged that VR training has enhanced practical competencies and decision-making skills, especially in resource-limited or high-risk environments. Our findings extend this literature by confirming not only the short-term benefits of VR training, but also its sustained impact over time, highlighting its potential to drive long-term behavioral change in disaster preparedness.

In contrast to prior investigations conducted primarily among first responders or health care professionals [38], our study surveyed community volunteers in rural Indonesia, a population that is frequently overlooked in disaster preparedness initiatives. By customizing VR content to the local environmental and cultural context, we made sure that the training was effective and engaging. These findings contribute to the existing literature by highlighting the need to culturally contextualize simulations and ensure community-specific design. The incorporation of localized situational scenarios that also feature geographical and architectural aspects is in accordance with best practices identified in other VR-based preparedness studies [40-42]. This aligns with VOICE-based disaster resilience training [43]; supporting knowledge acquisition is well-evidenced, and this study emphasizes the importance of demographic adaptations. The results also suggest the necessity of demographic customization in immersive digital interventions. Age and education level significantly moderated the effectiveness of the training, with older adults and those with lower educational attainment demonstrating less improvement. These findings reinforce the need for inclusive, age-sensitive instructional design within VR environments, acknowledging differences in cognitive processing, digital literacy, and user familiarity with technology. Previous research has largely overlooked these human factors in disaster training, making this an important area for future design optimization and research [40-42].

Moreover, our results suggest that VR should be incorporated into more comprehensive disaster readiness initiatives [44]. Although standalone VR modules may be useful, they do not truly prepare users for the highly nuanced and collaborative skills required in a real emergency. It would be better to integrate VR simulations with team-based drills and ethical dilemmas and decision-making tasks that better mirror the challenges of the real world and enhance preparedness. In Indonesia, VR simulations have used high-tech solutions with local community scenario use cases in educating communities about disaster mitigation. The success of engaging users, using interactive elements, and providing real-time feedback has been evidenced by success metrics such as anecdotes, success stories, and writing.

Although VR simulations clearly support individual knowledge acquisition, their true value lies in being part of a broader, more integrated disaster education approach. Instead of functioning in isolation, VR modules can be significantly more impactful when combined with practical components such as group-based emergency drills, scenario-based ethical problem-solving, and interactive role-play exercises that mirror real-life disaster conditions [44]. Such hybrid strategies provide a more authentic training environment, cultivating both individual readiness and team coordination in emergency contexts. In Indonesia, ongoing initiatives like those from the Asia Pacific Disaster Resilience Center have illustrated the potential for VR-supported training across multiple national settings. However, for such training to be truly effective and meaningful, the content must be grounded in the realities of local communities. This includes incorporating familiar environmental features, local architectural styles, and region-specific risk scenarios to ensure learners can relate the training to their everyday surroundings.

Despite the encouraging outcomes, there remain important barriers to widespread implementation, particularly in underserved rural areas. Many of these regions face persistent technological limitations, including inadequate access to high-speed internet, a shortage of VR equipment, and budgetary constraints that make advanced technology adoption difficult. To make VR-based education more inclusive, future programs should consider more affordable and accessible formats, such as smartphone-compatible VR apps, which can operate without sophisticated infrastructure. Although this study demonstrated positive changes in knowledge and preparedness over a 3-month period, ongoing evaluation is essential. Long-term follow-up studies are needed to examine how well participants retain information over time, how training influences real-life decision-making during emergencies, and whether periodic updates or refresher sessions are necessary. Insights from such research can guide the integration of VR tools into standard disaster preparedness curricula or community-based training programs in Indonesia and other countries with similar resource constraints.

### Limitations

This study, however, has several limitations despite its strengths. First, participants were not randomized into groups, making causal inferences using this quasi-experimental design difficult to interpret. Responses and information were collected cross-sectionally, which may introduce bias into the results. Second, the study was carried out in a defined country and culture, which might limit the applicability of the findings to a wider population. Although significant predictor variables for VR training effectiveness included age and education level, other demographic variables with potential influence on training outcomes, such as socioeconomic status, past disaster experience, and cultural background, were not explored in depth. VR technologies are highly adaptable and scalable around the world, which suggests the necessity of frameworks around further studies to include varied populations to test the sustainability of the technology across regions and within social spectrums. Third, although VR has been strongly promoted in this study as an affordable and readily available educational intervention, further consideration is needed on the possible barriers to implementation in low-resource settings. Barriers such as limited access to stable electricity, internet connectivity, technical support, and compatible hardware may mean that VR-based interventions are not always feasible, particularly in rural or underserved areas. If these initiatives are to benefit the underserved, it is important to build the right infrastructure to support their access and sustainability. Furthermore, the use of self-reported data for the knowledge and practices component may have led to a response bias. Future studies ought to include more objective measures to improve the validity, such as behavioral assessments or skill-based testing, rather than relying solely on self-report. Finally, the relatively short follow-up period of 3 months may have limited the ability to assess lasting retention and the behavioral impact of the intervention. Long-term follow-up periods and longitudinal studies are suggested to determine whether training effects are sustainable in the long run.

### Conclusion

Our study shows that VR simulation training is effective in increasing knowledge and self-reported practices toward earthquake preparedness in rural volunteers in Indonesia. Indeed, the impact occurring in this intervention group was maintained at follow-up, supporting the potential for immersive, scenario-led, dedicated VR learning in a group format. The study may be important in further underscoring the utility of incorporating VR into disaster preparedness programs, especially in rural areas where access to traditional training is limited. However, this should be interpreted with caution, as self-reported intentions to prepare may not result in behavioral change, emphasizing the need for additional strategies to encourage change in practice. Future work might examine whether these improvements in knowledge and perceived preparedness translate to actual behaviors, like stockpiling emergency kits or undertaking community drills. Additionally, VR scenarios could increase realism and effectiveness by including complex decision-making and responding to ethical dilemmas under stress. Our recommendations include conducting studies that examine the scalability, cultural adaptability, and long-term impact of VR-based disaster training with various populations.

## Abbreviations

VR: virtual reality
